# The American Dental Convention

**Published:** 1862-12

**Authors:** 


### THE AMERICAN DENTAL CONVENTION.

THIS body has pretty much become a New York institution; and we personally care very litte who runs the machine, so that it does good work. About two thirds of the members present at the late meeting reside in New York; and the consequence is the next meeting, like the last, locates in New York. And, as our New York brethren like to attend meetings of the profession when they are held in New York, it is very probably that New York will have quite a majority in the next meeting, and if so, it is just as probable that the following meeting will be in New York. Consequently, we have made up our mind that when we wish to attend a meeting of the Convention, we will go to New York; and we have concluded not to ask the brethren of New York to let the Convention leave New York; and if we have used the term New York in writing this, it was to work up our mind to an appreciation of the two leading ideas of our brethren of New York: first, that there is such a place as New York. Secondly, that there isn't any place besides New York. And in closing we wish to thank our brethren of New York, that though they will not let the Convention out of New York, yet they are always cordially willing to let the rest of us into New York. We wish also to assure our brethren of New York that we fully appreciate the power of New York, and the policy of New York, in the Convention; and lest they should still have doubts of our sincerity, we will add, *New York!* NEW YORK!! NEW YORK!!! W.

